# Transcriptomic analysis of zebrafish (*Danio rerio*) liver exposed to sediments from the Loa River

**DOI:** 10.1371/journal.pone.0356064

**Published:** 2026-10-09

**Authors:** Nataly Lobos-Parra, Natalia V. Leiva, Giovanni Larama, Mariella Rivas, Rodrigo Orrego

**Affiliations:** 1 Facultad de Ciencias del Mar y Recursos Biológicos, Laboratorio de Toxicología Acuática (AQUATOX), Instituto de Ciencias Naturales Alexander von Humboldt, Universidad de Antofagasta, Antofagasta, Chile; 2 Facultad de Ciencias del Mar y Recursos Biológicos, Programa de Doctorado en Ciencias Aplicadas mención Sistemas Acuáticos, Universidad de Antofagasta, Antofagasta, Chile; 3 Facultad de Ciencias Agropecuarias, Escuela de Medicina Veterinaria, Universidad del Alba, Antofagasta, Chile; 4 Facultad de Ciencias del Mar y Recursos Biológicos, Instituto de Ciencias Naturales “Alexander von Humboldt”, Universidad de Antofagasta, Antofagasta, Chile; 5 Biocontrol Research Laboratory, Universidad de La Frontera, Temuco, Chile; 6 Facultad de Ciencias del Mar y Recursos Biológicos, Laboratorio de Biotecnología Ambiental, Dpto. de Biotecnología, Universidad de Antofagasta, Antofagasta, Chile; Hainan University, CHINA

## Abstract

Metal(loid) contamination in aquatic ecosystems, particularly the Loa River in Chile, poses significant environmental and health challenges. This study investigated the molecular impacts of such contamination by exposing zebrafish (*Danio rerio*) to sediments from pre-impact and severely impacted zones of the Loa River, utilizing transcriptomic analysis of zebrafish liver. Sediment characterization confirmed arsenic as the main driver of contamination, with As concentrations significantly higher at La Finca. Our findings reveal a clear and distinct cascade of molecular responses in fish exposed to impacted sediments, which provides evidence for an Adverse Outcome Pathway. This AOP commences with Molecular Initiating Events, including significant differential expression of genes related to metal adsorption and homeostasis (*slc31a1*, *slc40a1*; FDR-adjusted q < 0.05; p < 0.05 for IT7 vs C0 and IT7 vs PIT7), signifying direct perturbations in intracellular metal regulation. This is followed by Key Events characterized by heightened cellular stress, evidenced by significant upregulation of *xbp1*, *xbp1a*, *xbp1b*, and *hsp90a/hspd1* (IT7 vs C0; p < 0.05), consistent with oxidative stress and endoplasmic reticulum dysfunction, as well as hypoxia-like conditions. Progressing further, significant differential expression of angiogenesis-related genes (*ptprb*, *mib1*, *adam10a*, *foxc1a*; p < 0.05) suggests activation of tissue remodeling processes. The cascade culminates in alterations of genes associated with early carcinogenic signaling, including epigenetic regulation (*hdac1*) and genomic instability (*rassf1*, *top2b*; FDR q < 0.05; p < 0.05). This research demonstrates the power of transcriptomics to differentiate the biological impact of contaminated sediments from pre-impact conditions, offering a detailed molecular fingerprint of sublethal effects crucial for identifying early warning signals of environmental degradation and understanding potential human health impacts.

## Introduction

Environmental contamination by heavy metals represents one of the most critical challenges for aquatic ecosystems globally, mainly due to their persistence, bioaccumulation potential, and inherent toxicity, even at low concentrations [1]. Although certain metals like Cu, Fe, Ni, and Zn are essential micronutrients, others, such as cadmium and arsenic, lack known biological functions and bioaccumulate in organisms, exerting toxic effects [2,3]. Once the metabolic, storage, and detoxification capacities are overwhelmed in aquatic organisms, the risk of toxicity increases [4]

The Loa River, located in northern Chile, is an example of an aquatic system severely impacted by industrial and mining activities [5]. These activities have been identified as responsible for increase environmental concentrations of metal(loid)s, with As being a notable contaminant [6,7]. Studies have reported metal concentrations in areas like La Finca (downstream area directly impacted by mining activity in the Loa basin) that significantly exceed international standards, while regions like Lequena (upstream preimpact area) show considerably lower levels, being considered as in-basin reference sites [8,9].

Previous studies on this particular extreme arid environment are scarce and have focused on determining the concentration of key metals in sediment, water, aeolian dust and some biological matrices [7,10], but not much in the dynamics and balances that develop between them. Physical, chemical and biological processes govern the availability of metals and their entry into food chains (biological matrix), remaining in suspension (air and water matrices), or precipitate to the sedimentary matrix, being retained and accumulated for long periods, especially in areas highly impacted by mining activity.

Metals can pose a risk to this river environment. Therefore, the assessment of metal bioaccumulation in aquatic model organisms, such as fish, is crucial for estimating trophic transfer and adverse effects, including those relevant to human health [11]. However, these assessments must go beyond mere concentration, encompassing internal bioavailability, metabolic pathways, and the mechanisms of toxic action involved [4]. It is fundamental to consider early sublethal effects at the molecular level and critical toxicological endpoints at the organismal level in order to improve risk assessment of metals in aquatic environments [1]. Metals, such as As, Cd, Cu, Pb and mixtures thereof can trigger complex toxic effects through various metabolic pathways including metal-binding proteins, regulated by transcription factors, that play a crucial initial role in cellular adaptation and protection [12], subsequently activates oxidative stress pathways, where key transcription factors induce the synthesis of detoxifying enzymes and antioxidant proteins [13] related to mechanisms that detect oxygen deficiency in cells [14] before hypoxia-induced angiogenesis [15], a crucial process in cancer progression [16], when its regulation is related to transcription factors that respond to hypoxia [17]. These complex carcinogenic pathways are very diverse and include DNA damage, oxidative stress, epigenetic modification, deregulation of cell proliferation and apoptosis, as well as immune system changes [18]. These disruptions activate specific cellular responses, including the regulation of pathways associated with detoxification, oxidative stress, and hypoxia [1], among others.

There is a growing recognition that the integration of transcriptomic level response is playing an important role in identifying organismal functional responses pathways to environmental stressors, providing evidence of for future specific biomarkers development. These techniques enable the identification of complete molecular networks and adaptive mechanisms, being vital for biomonitoring and understanding interactions at different levels of biological organization [19]. The integration of these molecular approaches is fundamental for a comprehensive understanding of metal toxicity and associated risks in the environment [2,20]. Regarding this, zebrafish (*Danio rerio*) is an ideal model for environmental toxicology and carcinogenesis assessment, due to its sequenced genome, high genetic homology with humans, sensitivity to chemical contaminants, and easy genetic manipulation [21,22]. This model fish has allowed for the exploration of how metal exposure alters oxidative stress indicators and the expression of various molecules [15].. The activation of cellular signaling pathways, such as the PI3K-Akt pathway, fundamental in human cancers, is widely studied in this organism for drug discovery [13].

Among vertebrate models, zebrafish (*Danio rerio*) stands out as a particularly well-suited organism for environmental toxicology because it combines strong practical advantages with deep evolutionary and physiological conservation relative to higher vertebrates, including humans. Its small body size, external fertilization, optical transparency of embryos and larvae, and high fecundity — clutches of up to 200 embryos every 7 days [23] — make it possible to maintain large experimental cohorts under tightly controlled laboratory conditions at a fraction of the cost of rodent studies [24]. Together with a fully sequenced genome [23] and low-cost maintenance amenable to high-throughput experimentation [24], these practical advantages accompany deep evolutionary conservation of vertebrate physiology, including xenobiotic-metabolizing pathways and stress-response mechanisms [24]. Moreover, zebrafish has been documented to display toxicity responses that are broadly analogous to those seen in mammalian biological models [23], which supports the translational relevance of findings obtained in fish. Importantly, at the genomic level it has been documented that approximately 70% of human protein-coding genes have at least one obvious zebrafish ortholog, and that this proportion rises to about 82% when restricted to human disease-related genes [22,25]. Furthermore, many of the developmental and signaling pathways underlying those diseases are conserved between zebrafish and humans [24], reinforcing the translational value of *D. rerio* as a model for studying health effects caused by environmental pollutants.

The high level of pollution reported in sediments of the Loa River and the lack of information regarding its potential effects on aquatic organisms, calls for a comprehensive investigation into the molecular impacts of metal(loid)s exposure in a model organism like the zebrafish (*Danio rerio*), in order to acquire the necessary knowledge for metal risk assessment. This study aims to integrate the transcriptomic responses of a widely used fish model exposed under controlled laboratory conditions to a complex sedimentary matrix, comparing an upstream unpolluted area with a severely impacted downstream area of the Loa River. By investigating the major molecular pathways responses in zebrafish to a metal contaminated sediment, we aim to propose potential initial biomarkers that will improve the identification and characterization of key events within the future Adverse Outcome Pathway (AOP) framework, for environmental management.

## Materials and methods

### Study site

The Loa River basin, situated in the northern Antofagasta Region, is one of the few limnic discharges into the Pacific Ocean in northern Chile, spanning approximately 440 km [26] (Fig 1). Originating at the northern base of the “Miño” Volcano, near the border between the Tarapacá and Antofagasta Regions [26], the river flows through a geochemically anomalous zone associated with significant porphyry copper deposits, including the Chuquicamata, El Abra, and Radomiro Tomic complexes [27]. This entire basin is profoundly affected by extensive anthropogenic activities, with mining operations being a primary concern [27]. These activities exploit copper deposits, leading to the dispersion of potential pollutants, both as solid particles via wind transport and dissolved components in the Loa River, which is a vital water source for domestic, agricultural, and livestock needs in this arid region [27,28].

**Fig 1:**
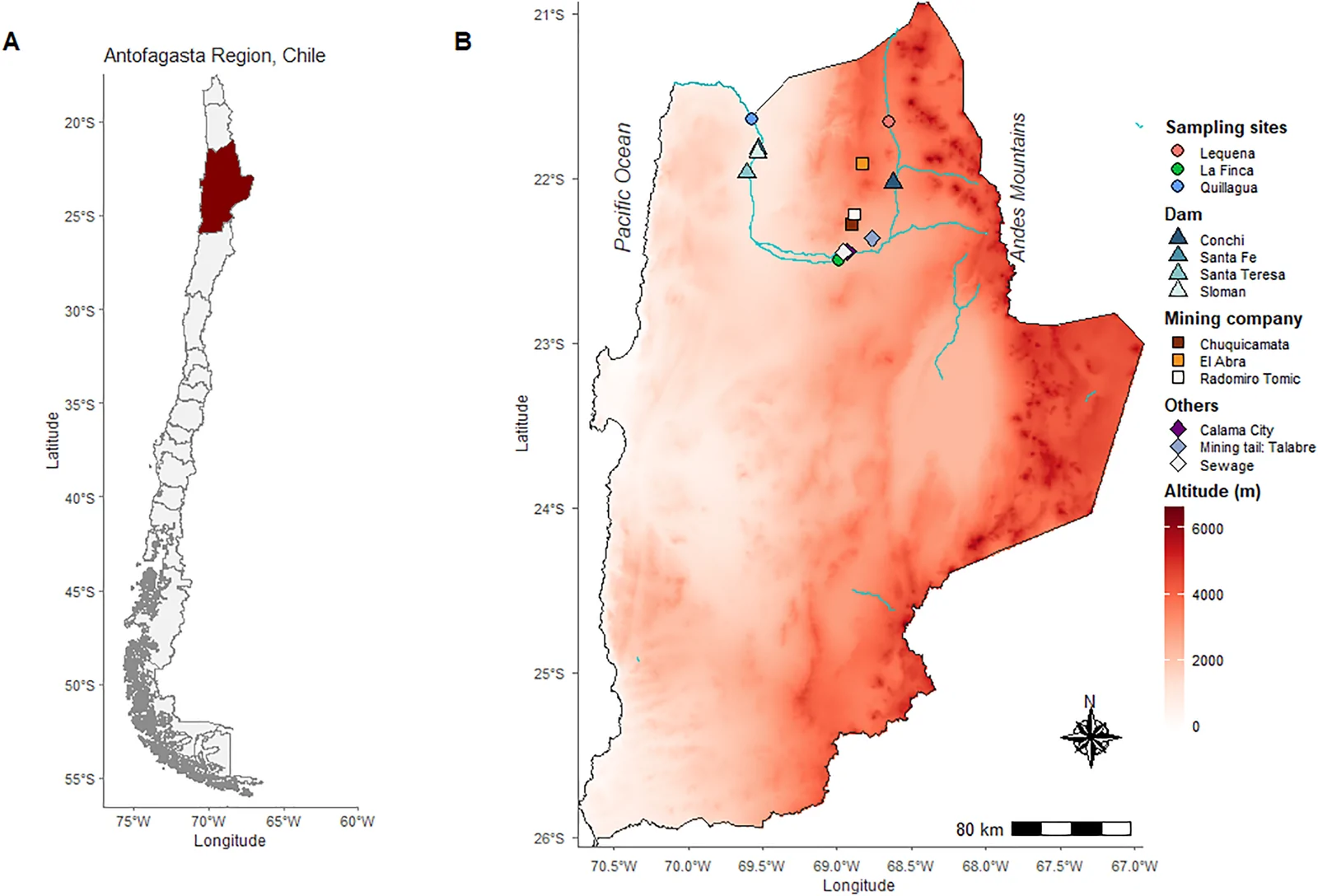
Study area in the Loa River basin, Antofagasta Region, northern Chile. (A) Location of the Antofagasta Region (Region II) within Chile. (B) Sampling sites (Lequena, La Finca, and Quillagua), dams, mining operations, and other relevant locations in the Loa River basin. Background shading represents elevation above sea level, with higher elevations shown in darker red tones. River networks are shown in blue. This figure was generated entirely in R from openly available geographic datasets and the authors’ own spatial data. Administrative boundaries were obtained from the open-source *chilemapas* package, elevation data from the *elevatr* package, and hydrographic features from OpenStreetMap contributors. No proprietary basemap or satellite imagery was used.

The main tributaries of the Loa River—the San Pedro, El Salado, and San Salvador rivers—all originate in the high Andes [29]. Since the early 20th century, the basin has undergone significant hydrological alterations due to the construction of three major dams: Sloman, Santa Fe, and Conchi [28]. These structures have not only regulated water flow but have also profoundly disrupted the natural transport of sediments from the Andes towards the Pacific Ocean [28]. Historically, reservoirs associated with these dams became heavily filled with sediments contaminated by mining waste from activities in the first half of the 20th century, with environmental laws for mining waste disposal only being implemented in 1994 [9]. Furthermore, the management of the Loa River’s water resources faces challenges due to a poor understanding of river-aquifer relationships, and upstream diversions have led to a significant reduction in river flow [30].

Mining activities throughout the region serve as a substantial source of toxic metals and metalloids, including arsenic [6] Studies have consistently revealed that metal concentrations in industrial-influenced areas like La Finca significantly exceed international environmental standards (Impact area), starkly contrasting with the considerably lower levels found in reference regions such as Lequena (Pre-Impact Reference area) [8,9].

### Sediment sampling

To investigate potential differences in metal(loid)s concentrations, and for subsequent laboratory bioassays, two representative sites were sampled, distributed altitudinally along the Loa River. These sites include: Lequena, located in the upper reaches of the river where potable water is collected for human consumption (Pre-impact zone), and La Finca, an agricultural area downstream of the city of Calama, highly affected by mining activity (Impact zone). Sediment samples were collected in triplicate and were kept refrigerated during collection and transport, finally stored at −80°C at the Aquatic Toxicology Laboratory (AQUATOX) of the University of Antofagasta, within 24 hours to ensure their integrity.

No special permits were required to collect sediment samples from the Loa River at the Lequena and La Finca sites. Both locations are publicly accessible riverbed areas not located within any protected natural reserve, national park, or privately restricted land. Sediment collection consisted exclusively of the retrieval of superficial substrate (top 5 cm) in triplicate, did not involve any biological matrix from protected or endangered species. None of the activities required authorization from wildlife, environmental, or mining regulatory authorities under Chilean national regulations.

For metal(loid)s analysis the < 63µm fraction of the sediment samples was used, because it is widely used in previous research as the fraction where contaminant metallic ions predominantly accumulate, allowing fair comparisons between samples with different overall grain size, be more bioavailable improving risk assessment [31]. After sieving, 400–600 mg of each sample (from riverbank sediment and sediment core types) were digested in teflon bombs. The digestion protocol involved 10mL HNO_3_ + 1mL HClO_4_ + 0.5mL HF in a MARS-X microwave digester (ICEM model 350), following the US-EPA 3051A procedure [32]. The concentrations of As, Cd and Cu in the digested sediment samples were analyzed by Atomic Absorption Spectrometry in a 6300 Shimadzu instrument. The accuracy and precision of the analytical methods were evaluated using the NIST 1646a estuarine sediment standards, provided by the National Research Council of Canada.

### Experimental conditions

Sexually mature wild-caught zebrafish (Danio rerio), obtained from a local pet store, were kept at the AQUATOX Laboratory. All experimental procedures were strictly approved by the Institutional Bioethics Committee of the University of Antofagasta (CEIC-UA, FOLIO N°: 372/2022) and comply with the standards of the Canadian Council for the Care of Animals. This certification was granted in 2022, it fully covered the chronological development of the project, including the sediment sampling in 2023 and the experimental bioassays carried out during 2023 and 2024. Furthermore, all research staff involved in the handling and sampling of the fish were specifically trained in animal care and welfare according to institutional and CCAC protocols [33,34]. The acclimation process was carried out for a period of 14 days prior to the start of the bioassay [35]. During this stage, the fish were kept in 70 L glass tanks at a density of approximately 5 fish/L in aerated, filtered (10^-5^ ^-2^ µm filters + activated carbon filter) [36,37], recirculated, and dechlorinated running tap water (28 ± 1 °C). Environmental conditions also included a natural photoperiod of 12–16 hours. Daily monitoring of pH, temperature, and dissolved oxygen levels was performed to ensure optimal conditions [35]. The fish were fed twice daily with commercial flake food (Tetraflakes® Pro) at a rate of 10% of their body weight per day, supplemented with *Artemia salina* nauplii produced *in situ* offered *ad libitum*, until the bioassay. To ensure the highest welfare standards, a rigorous daily supervision guideline was applied to evaluate the welfare and general condition of the animals before their transfer to the experimental units, following the ethical recommendations for the use of animals in research [35].

The two sediments used in the bioassay were collected in triplicate at each site and processed. Each experimental aquarium was loaded with 1 kg of wet natural sediment placed on the bottom, together with 10 L of the same filtered, dechlorinated tap water used during acclimation, yielding a sediment-to-water ratio of 1:10 of wet sediment per litre of overlying water [38]. Each sediment treatment (Pre-impact Lequena, PIT7, and Impact La Finca, IT7) was performed in duplicate aquaria (n = 2 per treatment), matching the triplicate design of the sediment sampling.

A total of 30 adult zebrafish (mean body weight 0.46 ± 0.15 g; mean total length 3.6 ± 0.41 cm) were randomly distributed among the experimental aquaria, with 7 fish per aquarium (i.e., 10 fish × 2 aquaria × 2 sediment treatments = 20 exposed fish total) and a fish density in the overlying water column of 1.6 fish L^-1^, computed from the number of fish divided by the overlying water volume [39]. A third group, C0 (control), consisted of 10 fish sampled directly from the acclimation tank on day 0, immediately before the bioassay started, and received no sediment exposure. Condition factor (Fulton’s K = W/L³ × 100, where W is body weight in g and L is total length in cm) was used as the primary endpoint to assess the general physiological status and health of the fish at sampling [40,41].

Fish were exposed for 7 days under semi-static conditions, with 30% of the overlying water renewed every 48 hours, following standard protocols for whole-sediment toxicity tests with fish [38]. Fish were not fed during the 7-day exposure to minimize particulate organic matter accumulation and prevent adsorption of metals to faecal material. Throughout the exposure period, the following water physico-chemical parameters were routinely monitored in each aquarium: dissolved oxygen ≥ 6 mg L^-1^, total NH3 ≤ 0.02 mg L^-1^, pH = 6.9 to 8.1, temperature = 28 ± 1 °C, all of which remained within the ranges recommended for zebrafish culture [39,42].

During the exposure period, the fish were fed commercial feed every two days at a rate of 3% of their body weight. Humane endpoints were established and monitored daily by trained staff using specific criteria, such as loss of swimming equilibrium, abnormal respiratory activity, or prolonged lethargy [43]. However, no animals died before meeting the criteria for euthanasia, and no acute lethality was anticipated for this sublethal study [43]. The euthanasia procedure was performed immediately (within 15 minutes) once animals reached the experimental endpoint under anesthesia (immersion in BZ-20: tricaine methanesulfonate) followed by cervical laceration, in accordance with the guidelines of the University’s Institutional Ethics Committee (FOLIO NO: 372/2022) and the standards of the Canadian Council for the Care of Animals [43,44]. The condition score was calculated based on the methods described by Clark et al. (2018) [42], typically using Fulton’s K factor to assess the physiological status and health of the fish.

### Tissue sampling and RNA extraction

Liver samples were collected in duplicate, pooling five livers per sample to ensure the minimum required RNA quantity for subsequent analyses. The pooled liver samples were preserved in RNA-Later solution (Sigma-Aldrich, USA) and stored at −80°C until RNA extraction. Total RNA was extracted in the Bioal Laboratory of the Faculty of Marine Sciences and Biological Resource (University of Antofagasta), using the miRNAeasy Mini Kit (Qiagen) following the manufacturer’s instructions. The extraction process included tissue homogenization in the lysis buffer provided by the kit, followed by RNA purification using silica membrane spin columns. RNA was eluted in RNase-free water, and its quantity and quality were assessed NanoDrop™ spectrophotometer (Thermo FisherScientific, United States), ensuring a 260/280 ratio between 1.8 and 2.0. RNA integrity was verified by electrophoresis on a 1% agarose gel.

### Library preparation and sequencing

Library preparation for sequencing was performed using the NEBNext Ultra II RNA Library Prep Kit for Illumina (New England Biolabs NEB, cat. no E7770S, United States). This process included mRNA isolation using polyA selection with magnetic beads (NEB, cat. no E7490S), followed by RNA fragmentation to generate appropriately sized fragments for sequencing. The fragmented RNA was reverse transcribed into complementary DNA (cDNA), and Illumina-specific adapters were ligated. The libraries were amplified by PCR under controlled conditions to minimize biases and ensure uniform sequencing coverage. Library quantification was conducted using fluorometry (Qubit 4.0, Thermo Fisher), and their quality was confirmed using a fragment analyzer (Bioanalyzer, Agilent Technologies). Sequencing was conducted at the Institute of Antofagasta, University of Antofagasta, using an Illumina MiSeq platform. The MiSeq Reagent Kit v3 (Illumina) was used, configured for paired-end reads with a length of 2x150 base pairs. This protocol enabled high-quality data generation for subsequent differential gene expression analysis of zebrafish liver samples exposed to sediments.

### Transcriptomic data analysis

Transcriptomic analyses were conducted using the KBase (DOE Systems Biology Knowledgebase) bioinformatics platform. Raw paired-end RNA-seq reads were subjected to quality control using integrated FastQC-based tools to assess sequencing quality, GC content, and read integrity. Adapter sequences and low-quality bases were removed during preprocessing to ensure high-quality reads for downstream analyses.

Filtered reads were aligned to the zebrafish reference genome (*Danio rerio, GRCz11* assembly) using the HISAT2 aligner, allowing accurate and efficient mapping of reads to the reference genome. Alignment quality was evaluated using BAM-level quality control metrics, including mean coverage, GC percentage, mapping quality, and insert size distributions, to ensure consistency among samples and experimental groups.

Gene expression quantification was performed using StringTie, which assembles transcripts and estimates gene-level expression. Expression values were normalized and reported as transcripts per million (TPM). TPM normalization accounts for both sequencing depth and gene length, enabling direct comparison of expression levels across samples and experimental conditions. TPM values were therefore used as the primary metric for quantitative expression analyses and data visualization.

For downstream analyses, TPM values were log_2_-transformed [log_2_(TPM + 1)] to stabilize variance and reduce the influence of highly expressed genes. Mean TPM values were calculated for biological replicates within each experimental condition: control at day 0 (C0), pre-impact at day 7 (PIT7), and impact at day 7 (IT7).

### Differential gene expression analysis

Differential gene expression analysis was performed using gene-level count data derived from the RNA-seq alignment and quantification process using *Deseq2*. Raw read counts were used as input for statistical testing, as count-based data preserves the discrete nature of sequencing reads and is required for robust inference of differential expression.

Genes were identified as differentially expressed genes (DEGs) based on comparisons between experimental time points (0 vs. 7 days) and between sediment exposure conditions (Pre-Impact vs Impact zones). Statistical significance was determined using model-based approaches implemented within the transcriptomic analysis workflow, with correction for multiple testing applied using the false discovery rate (FDR) method.

Genes were considered significantly differentially expressed when they met the following criteria: an adjusted p-value (FDR) below the defined significance threshold, and a biologically meaningful change in expression magnitude, expressed as log_2_ Fold of Change following criteria: |log 2 (Fold of Change)| > 1 and adjusted padj-value <0.05. The resulting DEG tables include gene identifiers, log_2_ Fold of Change values, standard errors, statistical test statistics, raw p-values, and FDR-adjusted q-values (S1 Table). These tables formed the basis for downstream functional interpretation and biological classification of transcriptional responses.

### Functional classification and visualization of transcriptomic patterns

Genes identified as differentially expressed were functionally annotated using curated databases, including *UniProt*, and classified according to their involvement in key biological processes relevant to environmental stress responses. Particular attention was given to genes associated with cancer-related processes, oxidative stress, and metal adsorption mechanisms, reflecting known molecular pathways affected by contaminant exposure.

To visualize global transcriptional patterns and relative expression changes across experimental conditions, heatmaps were generated using row-scaled z-scores calculated from log(TPM + 1) values (S1 Table). This approach emphasizes relative expression differences among conditions for each gene, facilitating the identification of coordinated transcriptional responses. Hierarchical clustering was applied to genes, while experimental conditions were displayed in a fixed temporal order (C0, PIT7, IT7). All statistical analyses and graphical visualizations were performed using RStudio [45], ensuring reproducibility and consistency throughout the analytical workflow.

## Results

### Spatial variation of metal(loid) concentrations in sediments

Metal(loid) concentrations measured in surface sediments of the Loa River showed clear spatial differences between the sampling sites of Lequena (Pre-impact) and La Finca (Impact) during 2023 (Fig 2). These sediments were subsequently used in the exposure experiments, and their chemical characterization was performed to establish a geochemical framework for interpreting the biological and transcriptomic responses observed in zebrafish.

**Fig 2:**
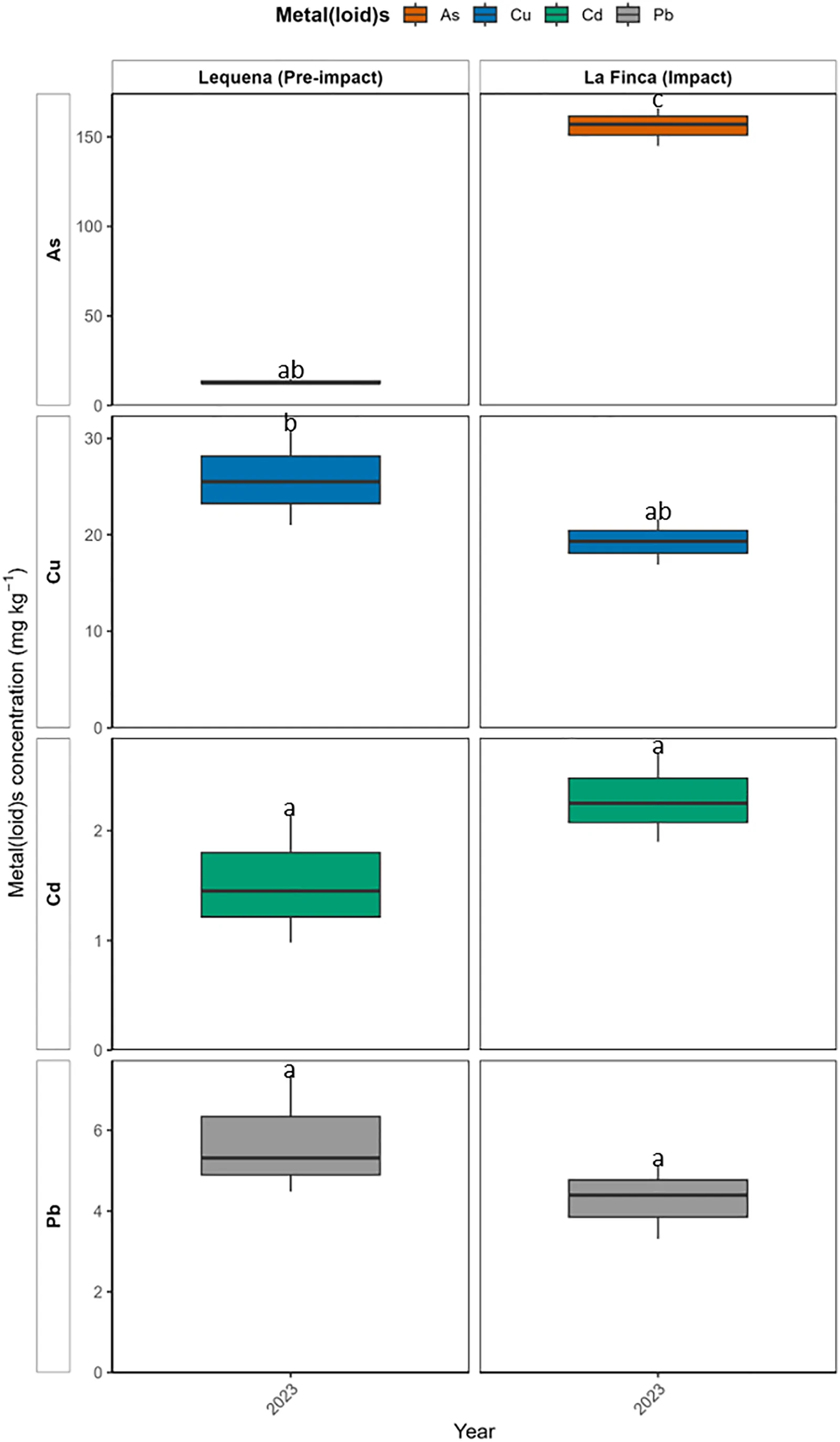
Metal(loid) concentrations (As, Cu, Cd, and Pb) in surface sediments of the Loa River during 2023, comparing the sampling sites Lequena (pre-impact), and La Finca (impact). Boxplots display the median (central line), interquartile range (box), and minimum and maximum values (whiskers). Letters above each box indicate statistically significant differences among sites for each metal, as determined by ANOVA followed by Tukey’s post hoc test (*p* < 0.05). Sites sharing at least one letter are not significantly different from each other.

Among the elements analyzed, As exhibited the most pronounced spatial gradient. Mean As concentrations were exceptionally lower in Lequena (13.2 ± 1.3 mg kg^-1^) and substantially higher in La Finca (156.0 ± 10.5 mg kg^-1^) (Table 1), representing an increase of more than an order of magnitude between pre-impact and impacted sites. Statistical analyses confirmed that As concentrations differed significantly between sites (ANOVA followed by Tukey’s post-hoc test, *p* < 0.05; Fig 2). This pattern indicates a strong spatial contrast associated with anthropogenic influence in the impacted section of the river.

**Table 1:** Mean, standard deviation (SD), maximum and minimum values (mg kg^-1^) of As, Cd, Cu and Pb in stream sediments of the Rio Loa Basin in 2023. Reference LC50 values (96 h) for zebrafish are also included.

Zone		As	Cd	Cu	Pb
*Lequena* *(Pre-impact)*	Mean	13,20	1,53	25,77	5,72
	SD	1,30	0,59	4,91	1,48
	Max	14,70	2,15	30,80	7,36
	Min	12,40	0,98	21,00	4,48
*La Finca* *(Impact)*	Mean	156,00	2,29	19,27	4,28
	SD	10,53	0,41	2,35	0,92
	Max	166,00	2,71	21,60	5,14
	Min	145,00	1,90	16,90	3,31
*LC50 (96h)*		17,50 ^a^	3,80 ^b^	0,21 ^b^	> 0,59 ^b^

Ref: ^a^ [46], ^b^ [47].

Cd, Cu, and Pb showed comparatively less spatial differentiation. Cu concentrations were slightly higher in Lequena than in La Finca, a pattern consistent with the geogenic background of Cu in the upper Loa River basin and its relatively lower enrichment in the impacted areas downstream. Cd concentrations were relatively homogeneous across sites, with only slightly higher values at La Finca and no statistically significant differences. Similarly, Pb concentrations did not differ significantly among sites, showing comparable levels along the analyzed spatial gradient (Fig 2).

To contextualize the 2023 sediment concentrations within a long-term pollution framework, they were compared with geochemical indices previously reported by Lobos-Parra et al. (2026) [48] (Table 2). These indices assess the degree of metal pollution relative to natural background; to distinguish between geogenic and anthropogenic sources of metals; integrates multiple elements to provide a comprehensive assessment of sediment, and an ecological risk indicator that estimates the probability of adverse biological effects associated with sediment-bound contaminants. Together, these indices provide a complementary and integrative perspective on sediment quality and ecological risk, allowing for temporal and spatial comparisons across the entire Loa River basin.

**Table 2:** Mean metal(loid) concentrations (mg kg^-1^) and associated sediment quality indices (Igeo, EF, PLI, and m-PEC-Q) at Lequena (pre-impact) and La Finca (impact) in the Loa River basin for the years 2014, 2015, 2017, and 2023, showing consistent spatial differentiation between reference and impacted sites [48].

		2014			2015		
		As	Cd	Cu	As	Cd	Cu
*Lequena* *(Pre-impact)*	**Mean concentration** **(mg Kg**^**-1**^)	24,50	0,50	80,48	15,37	0,50	38,57
	**Igeo**	Moderately polluted	Moderately polluted	Moderately polluted	Moderately polluted	Moderately polluted	Not polluted
	**EF**	No enrichment to minor enrichment	No enrichment to minor enrichment	No enrichment	No enrichment to minor enrichment	No enrichment to minor enrichment	No enrichment
	**PLI**	Low contaminated			Low contaminated		
	**m-PEC-Q**	21% risk			21% risk		
*La Finca* *(Impact)*	**Mean concentration** **(mg Kg**^**-1**^)	313,16	4,75	184,34	180,03	1,28	41,13
	**Igeo**	Heavily polluted	Moderately to heavily polluted	Moderately polluted	Heavily polluted	Moderately to heavily polluted	Not polluted
	**EF**	Significant to high enrichment	Moderate to significant enrichment	Minor to moderate enrichment	Significant to high enrichment	Moderate to significant enrichment	No enrichment
	**PLI**	Contaminated			Contaminated		
	**m-PEC-Q**	76% risk			76% risk		
		**2017**			**2023**		
		**As**	**Cd**	**Cu**	**As**	**Cd**	**Cu**
*Lequena* *(Pre-impact)*	**Mean concentration** **(mg Kg**^**-1**^)	15,24	0,11	17,34	13,20	1,53	25,77
	**Igeo**	Moderately polluted	Not polluted	Not polluted	Moderately polluted	Moderately to heavily polluted	Not polluted
	**EF**	No enrichment to minor enrichment	No enrichment to minor enrichment	No enrichment	No enrichment to minor enrichment	No enrichment to minor enrichment	No enrichment
	**PLI**	Low contaminated			Low contaminated		
	**m-PEC-Q**	21% risk			21% risk		
*La Finca* *(Impact)*	**Mean concentration** **(mg Kg**^**-1**^)	236,17	0,84	15,65	156,00	2,29	19,27
	**Igeo**	Heavily polluted	Moderately polluted	Not polluted	Heavily polluted	Moderately to heavily polluted	Not polluted
	**EF**	Significant to high enrichment	Moderate to significant enrichment	No enrichment	Significant to high enrichment	Moderate to significant enrichment	No enrichment
	**PLI**	Contaminated			Contaminated		
	**m-PEC-Q**	76% risk			76% risk		

Overall, these results demonstrate that As is the dominant geochemical factor differentiating pre-impact and impacted sites in 2023 and provides a solid environmental context for interpreting the transcriptomic alterations associated with sediment exposure in zebrafish.

### Experimental zebrafish condition

The condition factor indicated that both treated and control fish were in relatively good condition (values ~ 1.0) across all experimental exposures, except in La Finca (Impact) treatment (Fig 3). However, no significant statistical differences in condition factor (k) were observed between control and treatment zebrafish (ANOVA, *p* < 0.05).

**Fig 3:**
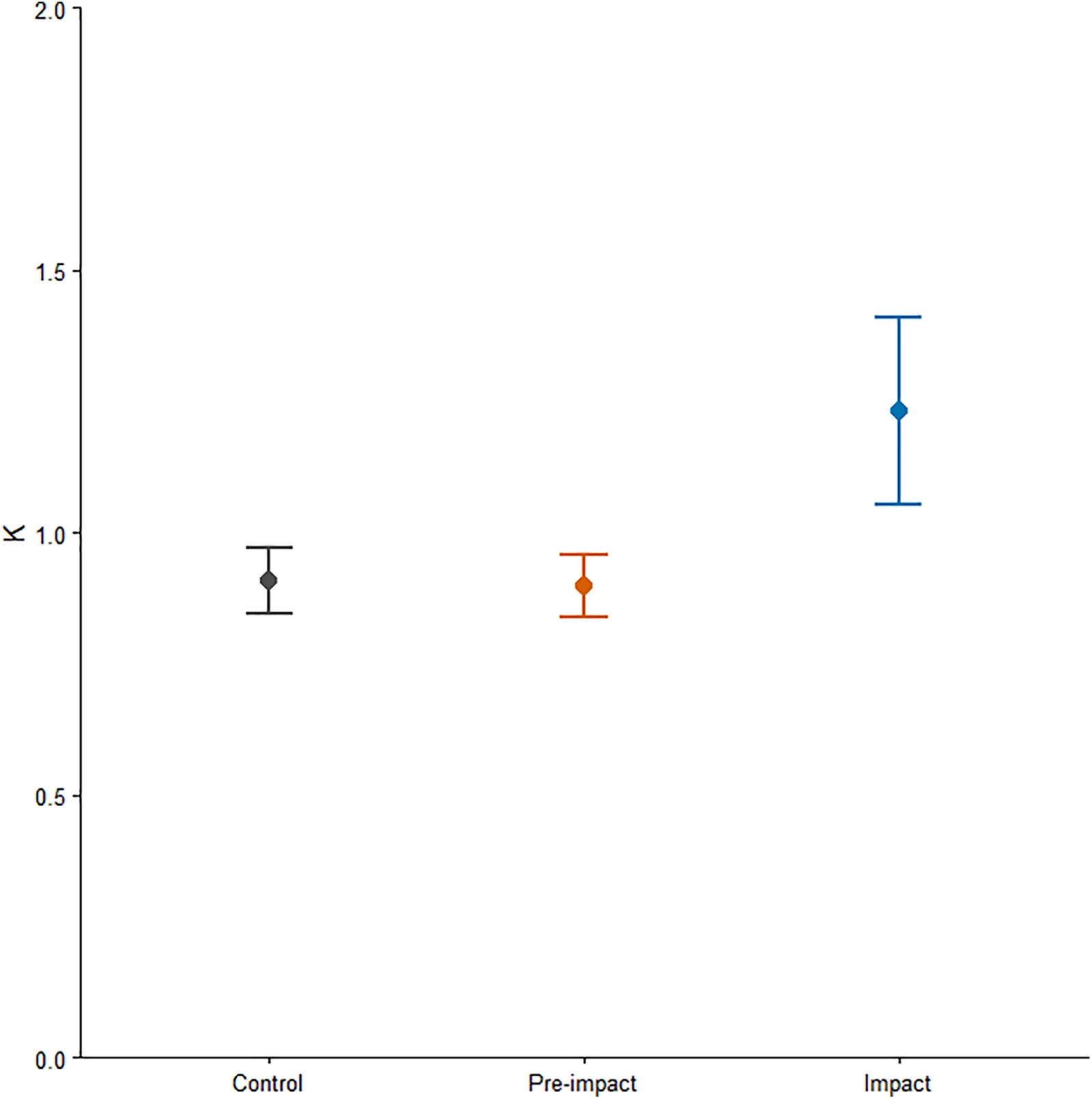
Least square means bi-dimensional plot representing the mean ± SD of the condition factor (K) of adult zebrafish against sediment treatments. The control group was sampled on day 0, and the Pre-impact (Lequena sediments) and Impact (La Finca sediments) treatments were sampled after 7 days of exposure.

### Sequencing and read mapping

The quality of the aligned RNA-seq data was evaluated using BAM-level quality control metrics generated within the KBase (DOE Systems Biology Knowledgebase) platform. Mean coverage values were comparable among samples within each experimental group (C0: Control, Pre-impact day 7: PIT7, and Impact day 7: IT7), indicating consistent sequencing depth across biological replicates (Table 3). GC content ranged between 40.46% and 44.89%, with no evidence of GC bias among treatments. Mapping quality scores were high across all samples, with mean values ranging from 8.61 to 20.90, supporting reliable alignment of reads to the *Danio rerio* reference genome. Insert size distributions were also consistent among replicates, suggesting uniform library preparation and sequencing performance. Collectively, these metrics indicate that the RNA-seq alignments were of sufficient quality and suitable for downstream differential expression and comparative transcriptomic analyses.

**Table 3:** BAM-level quality metrics of RNA-seq alignments. Mean coverage, GC percentage, mapping quality and insert size statistics for each sample across experimental conditions (C0, PIT7 and IT7), calculated from aligned BAM files. These metrics were used to assess alignment quality and data consistency prior to downstream expression analyses.

Sample	Group	Coverage means	Coverage sd	GC percentage	Mapping quality	Insert size
C01	Control T0	0.1227	1.1839	44.89	8.608	231
C02	Control T0	0.0428	0.5448	42.67	15.0337	207
PIT71	Pre-impact T7	0.0511	1.1398	44.13	12.1441	213
PIT72	Pre-impact T7	0.0485	1.3269	41.95	16.8248	211
IT71	Impact T7	0.0324	0.854	40.46	20.8987	212
IT72	Impact T7	0.0581	1.3826	42.47	16.1008	209

### Differential expression of metal-related genes

Transcriptomic analysis of zebrafish livers exposed for seven days to sediments from the Loa River revealed significant transcriptional alterations associated with metal uptake, transport, and homeostasis (Table 4; Fig 4). Exposure to impacted sediments (IT7) induced the differential expression of genes involved in metal handling, notably *slc31a1* (CTR1) and *slc40a1* (ferroportin), which play central roles in copper and iron transport, respectively. These transcriptional changes suggest altered intracellular metal availability and disruption of metal homeostasis under impacted conditions.

**Table 4:** List of identified genes of interest, classified according to their functional biological group (metal transport/adsorption, oxidative stress, angiogenesis, and carcinogenesis), used for the comparative analysis of gene expression across experimental conditions.

Gene	Comparison	Regulation in Impact	Main biological process	Biological group/ AOP stage
*slc31a1 (CTR1)*	CT vs IT; PIT vs IT	Downregulated	Copper uptake, metal homeostasis	**Metal transport/adsorption**
*slc39a1/ slc40a1*	CT vs IT	Altered (gene-specific)	Metal transport, cellular metal sensing	**Metal transport/adsorption**
*fbxl5*	PIT vs IT	Downregulated	Iron sensing, metal-regulated protein turnover	**Metal transport/adsorption**
*xbp1/ xbp1a/ xbp1b*	CT vs IT; PIT vs IT	Upregulated	ER stress, unfolded protein response	**Oxidative stress**
*hsp90a/ hspd1*	CT vs IT	Upregulated	Stress response, protein folding	**Oxidative stress**
*ptprb*	CT vs IT	Upregulated	Tyrosine phosphatase signaling, angiogenesis	**Angiogenesis**
*adam10a*	CT vs IT	Upregulated	Notch signaling, tumor progression	**Angiogenesis**
*tnfrsf1a*	CT vs IT	Upregulated	Inflammation, apoptosis-related signaling	**Angiogenesis**
*top2b*	CT vs IT	Upregulated	DNA topology, genomic instability	**Carcinogenesis**
*hdac1*	CT vs IT; PIT vs IT	Upregulated	Epigenetic regulation, chromatin remodeling	**Carcinogenesis**
*rassf1*	CT vs IT	Downregulated	Tumor suppressor, cell cycle control	**Carcinogenesis**

**Fig 4:**
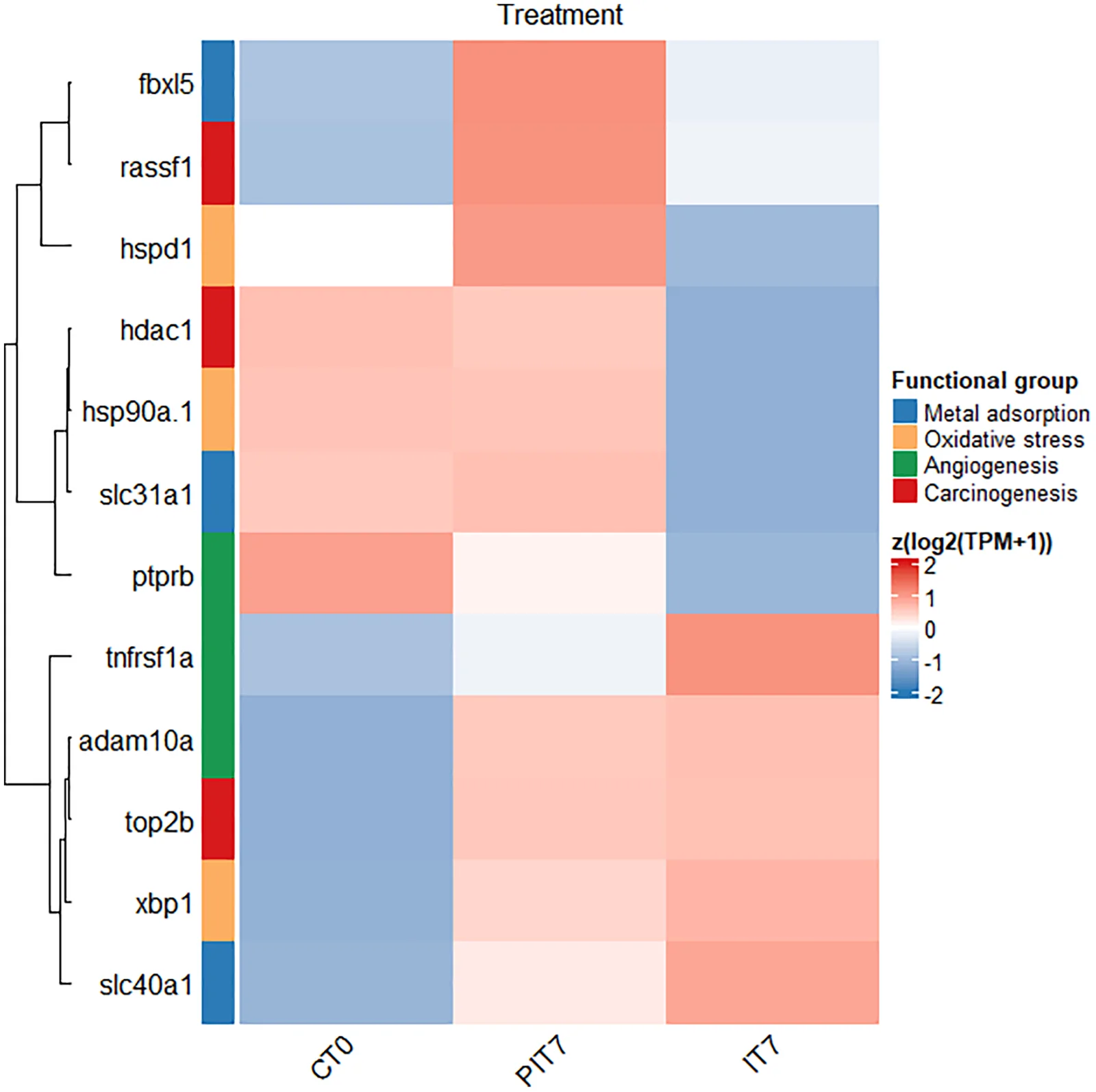
Heatmap of differential gene expression profiles among the experimental treatment groups: Control (CT0), Pre-impact sediment exposure, day 7 (PIT7), and Impact sediment exposure, day 7 (IT7). The heatmap includes genes associated with metal adsorption, oxidative stress, angiogenesis, and cancer-related processes. Gene expression values are represented as relative expression levels, with upregulated genes shown in red and downregulated genes shown in blue.

Consistently, modulation of *fbxl5*, a redox- and iron-dependent regulator involved in iron sensing and oxidative balance, was observed. The differential expression of *fbxl5* supports the presence of sustained perturbations in metal–redox equilibrium, likely driven by increased metal exposure in impacted sediments. The coordinated regulation of these genes highlights the sensitivity of hepatic metal-handling pathways to sediment-derived contaminants and indicates that metal exposure constitutes an early and central driver of the observed transcriptional responses.

Comparison between pre-impact (PIT7) and impact (IT7) treatments further revealed a persistent subset of metal-related differentially expressed genes, reinforcing the notion that impacted sediments induce sustained molecular responses rather than transient transcriptional fluctuations. These findings are consistent with the chemical characterization of impacted sediments, which exhibit elevated arsenic concentrations, and support a mechanistic link between metal exposure and downstream cellular stress responses.

### Oxidative stress and endoplasmic reticulum stress responses

Following alterations in metal homeostasis, a pronounced transcriptional response associated with oxidative stress and proteostasis was observed (Fig 4). Genes involved in cellular stress responses and protein quality control, including *hspd1*, *hsp90a.1*, *vdac1*, and *psmd7*, were differentially expressed in exposed treatments, indicating activation of adaptive mechanisms aimed at mitigating oxidative damage and maintaining mitochondrial and proteasomal function.

A particularly relevant finding was the differential expression of *xbp1*, a master regulator of the unfolded protein response and a key component of the endoplasmic reticulum (ER) stress pathway. *xbp1* exhibited increased relative expression in exposed conditions, with the highest levels observed in IT7, suggesting persistent ER stress under impact conditions (Table 4). The recurrent identification of *xbp1* as a differentially expressed gene in both control versus impact and pre-impact versus impact comparisons underscores its central role in mediating cellular adaptation to prolonged environmental stress.

The persistence of oxidative and ER stress-related transcriptional signatures suggests that zebrafish livers exposed to impacted sediments experience chronic cellular stress, likely driven by metal-induced reactive oxygen species production and disruption of protein folding homeostasis. These responses represent critical early events that may predispose cells to longer-term pathological outcomes.

### Cancer-related pathways (AOP Stages: Angiogenesis and Carcinogenesis)

In addition to metal and oxidative stress responses, exposure to impacted sediments induced significant transcriptional changes in genes associated with early carcinogenic processes, aligning with subsequent AOP stages.

Genes associated with angiogenesis and vascular remodeling were notably affected. *ptprb*, *adam10a*, and *tnfrsf1a* following exposure to impacted sediments. These genes, along with *mib1* and *foxc1a*, participate in endothelial signaling mediated by the VEGF–Notch pathways, which are essential for vascular development and remodeling. Their modulation suggests activation of tissue remodeling processes and inflammation, potentially reflecting adaptive or pathological responses to sustained cellular stress and precursors to pro-carcinogenic states [49–51].

The transcriptomic analysis also revealed alterations indicative of early carcinogenesis. *hdac1* (CT vs IT; PIT vs IT), involved in epigenetic regulation and chromatin remodeling, supporting potential epigenetic dysregulation. The persistence of *hdac1* across comparisons further supports the hypothesis that impacted sediments induce sustained epigenetic reprogramming [52]. Furthermore, genes related to cell proliferation control and genomic stability were affected, with *top2b*, crucial for DNA topology and genomic instability, showing increased expression. Conversely, *rassf1*, a known tumor suppressor involved in apoptosis and cell cycle control, exhibited decreased expression. These findings, along with the differential expression of *map3k4,* suggest activation of inflammatory and stress-related signaling pathways, contributing to the establishment of a pro-inflammatory tissue microenvironment.

Importantly, these molecular alterations do not imply neoplastic transformation within the experimental timeframe. Rather, they are consistent with early-stage molecular events that precede carcinogenesis, particularly under long-term, low-dose exposure scenarios typical of contaminated aquatic ecosystems*.*

## Discussion

Our transcriptomic analysis of zebrafish liver exposed to Loa River sediments revealed significant differential expression of genes. These findings delineate a clear molecular cascade, initiated by metal exposure, progressing through cellular stress responses, and culminating in pathways indicative of early carcinogenic processes. Crucially, these transcriptional alterations were distinct when comparing fish exposed to impact sediments versus those from the pre-impact zone, highlighting a site-specific biological response to environmental contamination.

The initial molecular event observed was the differential expression of genes involved in metal uptake and homeostasis, notably *slc31a1* and *slc40a1* [53,54]. These changes indicate direct perturbations in the intracellular regulation of metals specific to the contaminated environment, suggesting the organism’s immediate response to altered metal availability. The activity of metal transporters like *slc31a1* directly influences intracellular metal concentrations.

Following this initial interaction, our transcriptomic data show a activation of cellular stress pathways. The differential expression of the transcription factor *xbp1*, a central component of the endoplasmic reticulum stress response, alongside other genes associated with cellular stress and proteostasis, indicates significant cellular perturbation specifically in the impact group compared to the pre-impact conditions. Its activation can be triggered not only by hypoxia but also by oxidative stress and ER stress, conditions known to be exacerbated by metal exposure and evidenced in our data [55].

Further along this molecular cascade, the observed differential expression of angiogenesis-related genes, such as *ptprb*, *mib1*, *adam10a*, and *foxc1a* [49–51], strongly suggests the activation of HIF-1α-mediated pathways. These genes are pivotal in tissue remodeling and vascularization, providing molecular evidence of a progression from cellular stress towards processes indicative of angiogenesis, a key event in many pathologies, including cancer.

Finally, the transcriptomic profile in the impact zone also revealed alterations in genes associated with epigenetic regulation and early carcinogenesis, such as *hdac1*, *rassf1*, and *top2b*. This sequential activation of distinct gene groups—from metal handling to stress, angiogenesis, and cancer-related pathways—offers a critical window for early detection of toxicity progression before the development of overt cancer. In this context, toxicogenomics, as used in this study, is fundamental for deriving toxicological mechanisms and to potentially identify “early effect biomarkers” necessary to support the use of approaches such as the Adverse Outcome Pathways [20], providing a clear mechanistic representation of critical toxicological effects from initial molecular interaction to adverse outcome at the individual or population level.

### Arsenic as the main driver of molecular responses in Loa River sediments

This study integrates sediment geochemistry, transcriptomic profiling, and targeted gene expression analyses to evaluate sublethal biological responses induced by metal-contaminated sediments from the Loa River basin. Our results consistently identify arsenic as the primary element differentiating pre-impact and impacted sites, and as the main driver shaping the molecular responses observed in zebrafish liver. Sediments from La Finca Impact zone exhibit arsenic concentrations exceeding those of the Pre-impact site [8,9]. The river that carries this contamination is, in hydrological terms, the longest perennial drainage in northern Chile, crossing the Atacama Desert over approximately 440 km; its baseflow is overwhelmingly sourced from intra-arc aquifers located above 3,000 m elevation, and the confined Calama aquifer is recharged through Loa River infiltration between Conchi and Lasana [30,56]. This arsenic toxicity is a major toxicological problem worldwide [57], with wide-ranging effects on the health of living organisms [58], and is released by natural and anthropogenic sources [57], such as mining activities that severely impact the Loa River.

It is worth noting that the present experimental design specifically captured sublethal responses. Condition factor K was maintained at ~1.0 across all groups (ANOVA, p > 0.05; Fig 3), with all fish showing no mortality throughout the 7-day exposure. This result is consistent with the systematic review of Kroon et al. (2017) [40], which reported that the response of K to metal contamination is highly variable across fish species: among 12 reviewed studies, five showed no change, six showed a decrease, and only one showed an increase in K upon metal exposure. The authors concluded that K alone is not a specific biomarker of metal contamination and must be integrated with molecular endpoints to provide meaningful information about fish health [40]. The weight-to-length scaling coefficient was shown to discriminate yellow perch chronically exposed to Cd and Cu mixtures from reference fish in lakes of northeastern Ontario [59]. Together, these observations support the molecular AOP cascade detected in this study (MIE → KE1 → KE2 → AO) is a more sensitive and mechanistically informative endpoint than K alone for detecting early sublethal responses to As-rich sediments [40].

In fact, the arsenic enrichment detected in this basin has been documented over a decade of geochemical monitoring: from 2014 to 2023, the Geoaccumulation Index at La Finca consistently classified the site as “heavily contaminated” for arsenic, the Enrichment Factor indicated “very high enrichment” for this metalloid, and the mean Probable Effect Concentration Quotient (m-PEC-Q) gave a persistent 76% probability of toxicity at the same site across all sampled years [48]. This spatio-temporal continuity confirms that the arsenic load at La Finca represents a long-term sedimentary feature of the basin rather than an episodic event. Importantly, the trophic transfer of this arsenic has been quantified in situ: total As concentrations in algae and freshwater plants of the Loa River ranged from 20 to 341 mg As kg^-1^, with one Cladophora sp. sample hyperaccumulating 11,000 mg As kg^-1^ (approximately 1.11% of dry weight) and exhibiting a bioconcentration factor up to 13,910 relative to surface water, where inorganic arsenic species predominated [29].

As is a well-established environmental carcinogen in humans and other vertebrates, with a mode of action largely mediated by chronic oxidative stress, altered signal transduction, and epigenetic dysregulation, rather than direct genotoxicity [60]. This mechanism is consistent with the absence of acute toxicity or physiological impairment in exposed fish, together with the activation of molecular stress-response pathways observed in this study. Recent advances in fish transcriptomics support this interpretation. The comprehensive review of arsenic toxicology in fish and aquatic systems has documented that As consistently induces histopathological lesions in fish liver — vacuolation, apoptosis of hepatocytes, pyknosis, peliosis hepatitis, fatty infiltration and cirrhosis — at environmentally relevant concentrations, providing a phenotypic anchor for the hepatic transcriptomic signal that we report here [61]. Furthermore, a recent AOP-anchored transcriptome catalogue in zebrafish demonstrated predictive accuracies of 82.9–92.3% for several environmentally relevant metals [62], confirming that liver transcriptomics can quantitatively identify arsenic-specific molecular key events in vertebrate aquatic systems. Built upon this framework, our transcriptomic profiles reveal that As acts as the dominant MIE in the Loa River basin, and that it triggers a coordinated downstream cascade of stress and cancer-related transcriptional responses in the absence of overt somatic impairment.

Although Cu concentrations in our geochemical dataset also showed variation between sites, the spatio-temporal assessment of Lobos-Parra et al.(2026) [48] provides the key to interpreting this pattern: in their decade-long monitoring (2014–2023), Lequena consistently showed EF levels ranging from “no enrichment” to “minor enrichment” for Cu across all sampling years, whereas La Finca showed EF values from “no enrichment” to “moderate enrichment” for Cu only in 2023, and “very high enrichment” for As throughout 2014 and 2017 [48]. In other words, while As at La Finca is unambiguously anthropogenic, Cu is not: its enrichment at Lequena is geological — concentrations up to 80.48 mg/kg vs the global average soil value of 25 mg/kg — reflecting the naturally metal-rich lithology of the Loa River basin and the episodic sediment transport processes in the upper catchment [48]. This explains the apparent paradox of higher Cu at the reference site: the “Pre-impact” label at Lequena refers to anthropogenic As input specifically, not to a metal-free background; Cu at Lequena represents the natural geological baseline of the basin, whereas Cu at La Finca reflects this same baseline modulated by episodic dilution [48]. Consequently, the transcriptomic differences observed between PIT7 and IT7 cannot be attributed to Cu, since La Finca is not enriched in Cu relative to Lequena by anthropogenic input, supporting our interpretation that As — the only element showing “very high enrichment” at La Finca across years [48] — is the dominant MIE driving the hepatic molecular cascade documented here.

### Transcriptomic evidence of metal dysregulation and cellular stress

Our transcriptomic responses revealed an initial phase of metal dysregulation. The differential expression of genes involved in metal handling and intracellular homeostasis, including *slc31a1* [63], was significantly more pronounced in the impact zone samples compared to the pre-impact samples. This highlights a site-specific molecular perturbation in metal homeostasis within the hepatocytes as a direct consequence of arsenic exposure. Chronic arsenic exposure in zebrafish has shown alterations in metabolism and liver function and has been associated with increased *nr2f2*, *jun*, *k-ras*, and *apoE*, genes involved in oxidative stress and oncoprotein processes [64]. In support of this pattern, the broader toxicological literature documents that As exposure systematically disrupts normal biological functions in fish, with liver and kidney identified as the main target organs, where accumulation exceeds other tissues and results in disturbances of growth, reproduction, ion regulation, immune function, gene expression, enzyme activities, and histopathology [61].

Following metal dysregulation, a clear pattern of cellular stress emerged. Consistent with this interpretation, several genes associated with proteostasis and mitochondrial function [65] are indicated, indicating sustained cellular stress rather than transient adaptive responses. Similar molecular signatures have been reported in zebrafish and mammalian models exposed to arsenic and other metals, where prolonged oxidative stress activates unfolded protein response pathways and compromises mitochondrial function. When interpreted within a co-expression network analysis framework, the coordinated dysregulation of metal-handling genes (slc31a1, slc40a1, fbxl5) and stress-response genes (xbp1, hsp90a, hspd1) is consistent with the identification of discrete gene modules whose eigengenes correlate with treatment, supporting the biological coherence of the molecular cascade described here and providing a methodological framework for future integrative biomarker discovery across the Loa basin.

A central finding of this study is the differential expression of *xbp1*, a master regulator of the UPR. XBP1 activation reflects a conserved adaptive mechanism that supports cell survival under chronic stress conditions. In cancer biology, XBP1 has been shown to promote cellular adaptation to hostile microenvironments characterized by oxidative stress and metabolic limitation, linking environmental stressors to carcinogenesis-related pathways [60]. This activation, linked to mitochondrial dysfunction and ER stress, can be an important factor for HIF-1α regulation [66], thus connecting environmental stress with pro-carcinogenic pathways. Our transcriptomic observation of *xbp1* upregulation, particularly in the impact zone samples, provides strong molecular evidence of chronic cellular stress potentially contributing to a pro-carcinogenic environment and signaling a transition towards hypoxia-related responses.

### Angiogenesis and early carcinogenesis-related pathways

Building upon the observed cellular stress, our transcriptomic analysis also identified significant differential expression in genes associated with angiogenesis and early carcinogenesis pathways, prominently in the impact zone.

Several genes identified in the transcriptomic analysis as differentially expressed between the pre-impact and impact zones are directly associated with epigenetic regulation and genome stability. The modulation of *hdac1* observed in this study supports the hypothesis that chromatin remodeling constitutes an early molecular response to chronic arsenic exposure [52]. Arsenic exposure can induce epigenetic modifications, such as DNA methylation, which have been shown to influence the regulation of gene expression and are closely related to human health. Furthermore, the differential expression of genes such as *rassf1* and *top2b* in the impact group reinforces the indication of altered control of cell proliferation and genomic stability, suggesting the initiation of pathways relevant to early carcinogenesis specifically in the contaminated environment. Our observation of significant epigenetic response (hdac1 upregulation in IT7) in the absence of overt phenotypic effects is consistent with the broader mechanistic understanding that arsenic is a potent epigenetic modifier. The zebrafish-specific review of Aluru (2017) [67] emphasizes that environmentally relevant arsenic exposure can induce DNA methylation changes, histone modifications, and noncoding RNA dysregulation, all of which can precede tumorigenesis [67]. Likewise, the literature on global histone post-translational modifications under arsenic exposure indicates that arsenic causes a global loss of H4K16ac — a hallmark of most human cancers — across four studies using AsIII and MMAIII at doses of 0.2–10 µM [68]. This provides a direct mechanistic link between the hdac1 signal detected in our IT7 group and the well-established arsenic–epigenetic axis [67,68].

Crucially, genes associated with angiogenesis and vascular remodeling, including *ptprb*, *mib1*, *adam10a*, and *foxc1a* [49–51], were significantly differentially expressed in the impact-exposed zebrafish. These genes are key players in endothelial signaling and vascular development, and their modulation suggests activation of tissue remodeling processes and angiogenesis, often indicative of chronic stress and precursors to pro-carcinogenic states.

Importantly, these molecular alterations do not imply neoplastic transformation within the experimental timeframe. Rather, they are consistent with early-stage molecular events that precede carcinogenesis, particularly under long-term, low-dose exposure scenarios typical of contaminated aquatic ecosystems.

### Proposed AOP: From metal exposure to carcinogenic signaling

Our study proposes an Adverse Outcome Pathway that integrates a cascade of transcriptomic changes observed in zebrafish liver, linking metal exposure in Loa River sediments to early carcinogenic signaling (Fig 5). This AOP is fundamentally supported by the distinct gene expression profiles detected when comparing pre-impact and impact zones.

**Fig 5:**
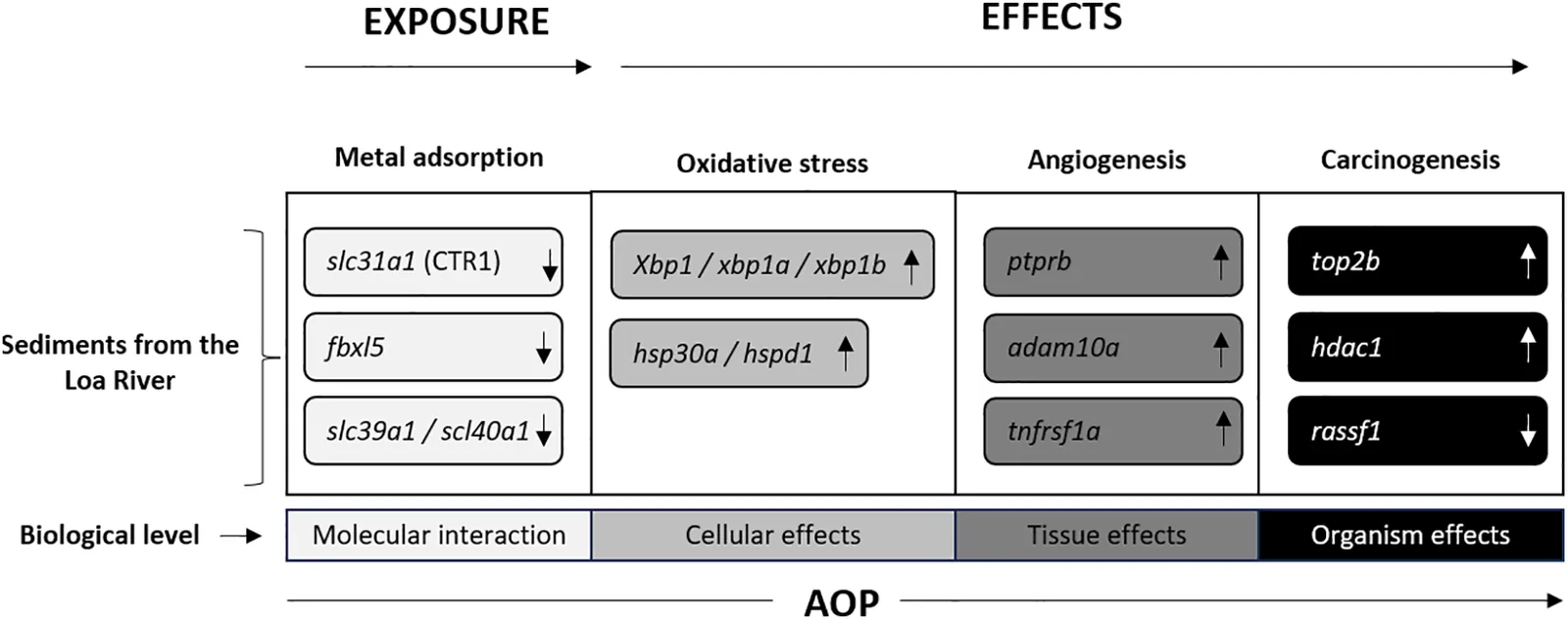
Proposed adverse outcome pathway (AOP) linking exposure to sediments from the Loa River to early carcinogenic signaling in zebrafish. Bioavailable metals perturb metal transporters, resulting in oxidative stress and hypoxia-related responses. These processes promote angiogenic signaling and converge on transcriptional alterations associated with carcinogenesis which could represent key integrative biomarkers connecting metal adsorption and oxidative stress within cancer as final adverse outcome.

The initial molecular initiating event is characterized by the differential expression of genes involved in metal adsorption and homeostasis, such as *slc31a1* and *slc40a1* [53,54]. These alterations reflect the organism’s direct interaction with bioavailable metals, particularly arsenic, leading to perturbations in intracellular metal balance.

This Molecular Initiating Event (MIE) then triggers key events related to cellular stress. Our transcriptomic data, specifically the upregulation of *xbp1* and other genes associated with proteostasis and mitochondrial function [55,65], indicate significant oxidative stress and ER stress. This cellular distress is consistent with an environment promoting a hypoxia-like state.

Progressing from cellular stress, a subsequent key event involves angiogenesis. Even though the gene that regulates HIF-1α was not overexpressed in our transcriptomic analysis, the widespread and significant differential expression of its downstream targets and related pathways, such as the angiogenesis-related genes *ptprb*, *mib1*, *adam10a*, and *foxc1a* [49–51], strongly implicates HIF-1α activation [60]. These genes are crucial for vascular remodeling, clearly differentiating responses between pre-impact and impact treatments, and signifying a critical step towards pro-carcinogenic processes.

Although HIF-1α itself was not directly overexpressed, the widespread modulation of its downstream targets strongly implicates its activation. Importantly, this apparent transcriptomic paradox is the expected molecular signature of HIF-1α pathway activation: HIF-1α is canonically regulated at the post-translational level rather than at the transcriptional level, so HIF-1α mRNA abundance can remain essentially unchanged while HIF-1α protein rapidly accumulates [69]. Under normoxic conditions, HIF-1α prolyl hydroxylases (PHDs/EGLNs) hydroxylate two conserved proline residues within the oxygen-dependent degradation domain of HIF-1α using O_2_ as an obligatory co-substrate; the hydroxylated protein is then recognized by the von Hippel-Lindau (pVHL) E3 ubiquitin ligase complex and rapidly degraded by the 26S proteasome [70]. Under metal-induced stress, including arsenic exposure, PHD activity is inhibited — directly through enzyme inhibition and indirectly through disruption of the Fe²⁺ and ascorbate cofactors required to maintain PHD catalytic iron in its reduced, active Fe² ⁺ state [71,72] — allowing HIF-1α to escape proteasomal degradation, accumulate, translocate into the nucleus, dimerize with HIF-1β/ARNT, and bind hypoxia response elements in target gene promoters [73,74]. Arsenic has been documented to stabilize HIF-1α via ROS-dependent PHD inhibition in mammalian cells [13], and to modulate the angiogenic program in zebrafish embryos [75]. The entire cascade operates at the protein-stability level, so HIF-1α protein can rise markedly within hours while HIF-1α mRNA abundance remains essentially unchanged [69]. The coordinated upregulation of HIF-1α downstream targets observed here is therefore the transcriptomic read-out of HIF-1α protein stabilization rather than an mRNA-level induction, and integrates coherently into the proposed AOP cascade as a mechanistic link between arsenic-driven perturbation of the PHD-VHL-proteasome axis and the pro-angiogenic transcriptional program detected in zebrafish liver.

Finally, our AOP culminates in molecular changes indicative of early carcinogenic signaling, including epigenetic regulation and altered genomic stability. The differential expression of genes like *hdac1* [52] for epigenetic modifications, and *rassf1* and *top2b* for cell proliferation control, directly links the prior events to a pro-carcinogenic environment. AOPs can be an efficient approach to provide a causal link between molecular responses [76], manifested at higher levels of biological organization.

In summary, the transcriptomic differences between pre-impact and impact conditions provide robust molecular evidence for this proposed AOP: metal adsorption (*slc31a1*) leading to oxidative stress/hypoxia (*xbp1*), which then promotes angiogenesis (*ptprb*), eventually contributing to a carcinogenic outcome (*hdac1*) (Fig 5). These specific molecular changes observed in the transcriptome serve as strong indicators for the identification of future integrative biomarkers connecting metal detoxification and oxidative stress within cancer as a final adverse outcome.

Importantly, the structure of the AOP proposed here — MIE (metal dysregulation) → KE1 (oxidative/ER stress) → KE2 (angiogenesis-related signaling) → AO (early carcinogenic signaling) — is consistent with the recent AOP-anchored zebrafish transcriptome catalogue of Chen et al. (2024) [77], which demonstrated that AOP-based molecular key event networks can identify the molecular key events by which metals such as arsenic produce coordinated biological responses. This convergence between our empirically derived zebrafish AOP and this computationally anchored framework supports the broader applicability of our proposed pathway for ranking As-contaminated sediments within regulatory AOP networks.

From a methodological standpoint, our AOP proposal is consistent with the molecular AOP framework recently formalized by Martens et al. (2023) [78]. In that work, connections between KEs of the AOP-Wiki and curated biological pathways in WikiPathways were defined using BridgeDb and integrated into Cytoscape, providing a method for the analysis and interpretation of transcriptomics data to identify KE activation. The authors validated the framework through case studies on liver steatosis and mitochondrial complex I inhibition, and emphasized that the OECD has recently published a formal reporting framework for transcriptomics and metabolomics technologies in risk assessment applications [78]. They conclude that the extension of AOPs with molecular markers, gene sets, or pathways would be an essential step towards the integration of high-throughput transcriptomics data into risk assessment studies [78]. The same authors highlighted that quantitative AOPs, developed from qualitative AOPs to provide quantitative descriptors for KEs and KERs, can serve as predictors of adversity [78], a perspective that aligns with our empirical use of empirically named KEs (MIE, KE1, KE2, AO) derived from the differential expression table in zebrafish liver.

The construction of our AOP is also conceptually aligned with the transcriptomics-to-AOP work of Wiklund, Wincent and Beronius [79], which performed RNA-Seq on 5-day-old zebrafish embryos exposed to Cd and PCB126 by linking transcriptional profiles to an estrogen-androgen-thyroid-steroidogenesis AOP network via Gene Ontology Biological Process terms, supported by Enrichment Maps in Cytoscape and QIAGEN Ingenuity Pathway Analysis. That work evidenced that the EATS-related pathways are highly conserved across vertebrate species and there is good empirical support for read-across between fish and mammals [79]. The cross-model convergence between embryonic Cd/PCB126 exposure and our adult sediment exposure in the same species reinforces zebrafish as a robust vertebrate platform for AOP-anchored risk assessment of mining-impacted freshwaters [79].

The AOP framework proposed here is built entirely on transcriptomic evidence, and no histopathological anchoring of the molecular KEs was conducted in the present study. Although this approach is consistent with the recent literature on AOP-anchored transcriptomics catalogues — which have achieved 92.3% experimental accuracy across multiple chemicals tested in zebrafish [77] — it should be noted that mRNA changes do not always correlate with protein-level or phenotypic outcomes [80], and that anchoring such responses to adverse phenotypes arising after chronic exposure remains a critical validation step [81]. Future work should therefore include histopathological evaluation of hepatic and vascular tissue, complemented by proteomic analysis, to bridge the molecular KEs identified here with the cellular and organismal AO. Interestingly, the only proteomic counterpart performed to date in this specific exposure scenario identified lipid metabolism/transport as the most perturbed pathway in zebrafish liver after 7-day exposure to 50 ppb sodium arsenite [82], providing cross-omic corroboration of the oxidative metabolic dysregulation we documented. This converging evidence supports — but does not replace — the need for the histopathological validation that we now propose as the natural next step of this research line.

### Environmental and human health implications

The molecular responses identified in zebrafish exposed to As-rich sediments from the Loa River basin have important implications for both environmental monitoring and human health risk assessment. The activation of pathways related to metal dysregulation, oxidative stress, proteostasis imbalance, epigenetic modulation, and tissue remodeling highlights the potential for chronic, low-level arsenic exposure to induce biologically meaningful effects long before overt toxicity becomes apparent [60]. Our transcriptomic data provides a detailed molecular fingerprint that clearly differentiates the biological impact of contaminated sediments from the pre-impact conditions, offering an early warning system for environmental degradation.

Given the high degree of genetic and physiological conservation between zebrafish and other vertebrates, including humans, the molecular signatures observed here are relevant beyond the experimental model. In particular, the predominance of arsenic-driven responses supports concerns regarding long-term exposure risks for aquatic organisms and human populations relying on water resources within the Loa River basin [8]. Given the high genetic homology with humans [83] and that zebrafish is widely used in translational toxicology to evaluate chemical effects and their conservation in different systems, the molecular signatures observed here are highly relevant for extrapolating potential human health impacts.

At a broader scale, these findings align with a recent systematic review of in situ fish transcriptomic studies, which identified 38 commonly dysregulated genes associated with lipid and xenobiotic metabolism, oxidative stress, hormonal signaling and immune/inflammatory pathways across multiple fish species exposed to environmental contamination [84]. The authors of that review explicitly highlighted that such studies, including those using zebrafish in mining-impacted river systems, are critically underrepresented in South America and that transcriptomic profiling of resident or sentinel fish exposed in situ or ex situ is an essential tool for identifying region-specific molecular biomarkers of contamination [84]. Our work directly addresses this gap by providing the first transcriptomic fingerprint of sublethal responses to As-contaminated sediments in the Loa River, the most mining-impacted basin in northern Chile. Recent studies on the sustained effects of developmental arsenic exposure in zebrafish reinforce this conclusion, demonstrating that arsenic-induced transcriptional changes can persist well beyond the exposure window [85] — an observation that increases the public health significance of chronic exposure to sediments from arsenic-enriched mining basins.

At the bacterial community level, Zárate et al. (2020) [86] documented that bacterial diversity in the Loa River gradually decreases from headwaters to the lower reaches, with the Shannon index decreasing from 5.95 upstream to 3.92 in the middle section and 3.85 in the lower reaches, and documented high abundances of bacterial communities associated with polluted water in the middle section near Calama [86] — independent physicochemical evidence of anthropogenic stress in the same river sector in which our chemical detection of 12-fold arsenic enrichment at La Finca was made. This microbial-plus-transcriptomic convergence on a single spatial gradient reinforces the sensitivity and biological relevance of hepatic zebrafish transcriptomics as an early signal of cumulative metal pollution in arid watersheds.

The ecological plausibility of these findings is further supported by Ríos-Escalante et al. (2023) [87], who documented that the benthic macroinvertebrate community composition in the Loa after the Salado River confluence was significantly different from those in the Salado River and in the Loa before the Salado confluence, with the salinity increase after the Salado junction — 5 g/L in the Salado — being a primary structuring factor. The same river system has been characterized microcrustacean-wise by De Los Ríos et al. (2010) [88], who reported that species richness in the Loa was significantly and positively correlated with altitude (r = 0.610; P = 0.041), with high-altitude sites harboring the amphipods *Hyalella fossamanchini* and *Hyalella kochi* and low-altitude sites hosting only the ostracod *Heterocypris panningi* [88]. Together, these ecological signals indicate that the same upstream-to-downstream contamination gradient documented here at the chemical level is also detectable at the bacterial invertebrate community level, reinforcing the ecological significance of our transcriptomic biomarker fingerprint.

Finally, the integrative biomarker work of Salem et al. (2025) [89], who studied *Clarias gariepinus* exposed to metal contamination in the Kitchener Drain and found that significantly elevated hepatic expression of genes related to metal toxicity, oxidative stress, inflammation, heat shock response, and xenobiotic metabolism collectively constitutes an adaptive response involving multiple stress-response pathways, showing how molecular biomarkers serve as early warning signals for water pollution [89], complements our findings and supports the value of an integrative biomarker framework in which transcriptional biochemical and histological endpoints are jointly applied to detect early biological responses before overt toxicity manifests.

Overall, this study demonstrates the value of integrating chemical characterization with transcriptomic analysis to detect early warning signals of environmental stress. Such approaches are especially relevant in arid and highly mining-impacted regions, where chronic exposure to arsenic and other metals represents a persistent challenge for ecosystem integrity and public health. It is important to emphasise that the transcriptomic signature reported here represents the integrated molecular response of adult zebrafish liver to the field-collected, multi-contaminant sediments of the Loa River basin, in which As, Cu, Cd and Pb are known to co-occur in the < 63 µm fraction of the sediments at the same sites investigated [48]. Our characterisation of the sediments does not allow us to deconvolve the contribution of each co-occurring chemical to the gene-expression profile; the proposed adverse outcome pathway is therefore framed around arsenic as the dominant chemical stressor within a realistic exposure mixture, not as a single-stressor causation. This qualification is consistent with the use of whole-sediment ecotoxicological bioassays, which are designed to capture environmental realism rather than isolate the response to any single chemical [90].

## Conclusion

This study utilized transcriptomic analysis in zebrafish liver exposed to Loa River sediments to elucidate the molecular impacts of chronic metal contamination, particularly arsenic. Our findings reveal a clear and distinct molecular cascade initiated by exposure to contaminated sediments from the impact zone compared to the pre-impact zone. This cascade provides robust transcriptomic evidence for a proposed Adverse Outcome Pathway linking metal exposure to early carcinogenic signaling.

The AOP commences with Molecular Initiating Events characterized by the differential expression of genes involved in metal adsorption and homeostasis, such as *slc31a1* and *slc40a1*. These transcriptional changes reflect direct perturbations in intracellular metal regulation, signifying the organism’s immediate response to bioavailable arsenic in the environment. Following this initial interaction, our data indicate the activation of key events related to cellular stress, evidenced by the upregulation of *xbp1* and other genes associated with proteostasis and mitochondrial function, consistent with oxidative stress and a hypoxia-like state.

Further along this pathway, the significant differential expression of angiogenesis-related genes, including *ptprb*, *mib1*, *adam10a*, and *foxc1a*, underscores the progression from cellular stress to processes indicative of tissue remodeling and angiogenesis. Although HIF-1α itself was not directly overexpressed, the widespread modulation of its downstream targets strongly implicates its activation as a critical step. Finally, the AOP culminates in molecular changes indicative of early carcinogenic signaling, with alterations in genes associated with epigenetic regulation (*hdac1*) and genomic stability (*rassf1, top2b*).

In summary, this research demonstrates the power of transcriptomics to differentiate the biological impact of contaminated sediments from pre-impact conditions, providing a detailed molecular fingerprint of sublethal effects that precede overt toxicity. This approach is crucial for identifying early warning signals of environmental degradation and for developing integrative biomarkers within an AOP framework. Given the genetic conservation with humans and the high relevance of zebrafish as a translational model, these findings have significant implications for environmental risk assessment and understanding potential human health impacts in regions affected by mining activities like the Loa River basin.

Finally, our application of the AOP-anchored transcriptome framework reinforces the value of zebrafish as a translational model for systematically linking metal exposure to carcinogenic signaling in mining-impacted arid regions, particularly along the Loa River basin, and supports the urgent need for region-specific transcriptomic monitoring programs in South American watersheds where such data remain scarce.

## Supporting information

S1 Table Minimal dataset for the transcriptomic analyses. The supporting data are organized into four worksheets: (1) the matrix used to generate the Figure 4 heatmap, including mean TPM values and the corresponding row-wise z-scores for C0, PIT7, and IT7; (2) differential expression results for the C0 vs. IT7 comparison; (3) differential expression results for the PIT7 vs. IT7 comparison; and (4) the complete TPM expression matrix for the six RNA-seq samples, comprising two biological replicates per treatment (C0, PIT7, and IT7).
(XLSX)
